# OnabotulinumtoxinA in Resistant Depression: A Randomized Trial Comparing Two Facial Injection Sites (OnaDEP Study)

**DOI:** 10.1155/2024/1177925

**Published:** 2024-09-12

**Authors:** Caroline Ceolato-Martin, Claire Chevallier-Collins, Jean-Pierre Clément, Eric Charles, Aurélie Lacroix, Danièle Ranoux

**Affiliations:** ^1^Esquirol Hospital, University Centre for Adult and Elderly Psychiatry and Addictology, 15 Rue du Dr Raymond Marcland Limoges, Limoges 87000, France; ^2^Private Practice, 8 bis Rue Victor Hugo La Rochelle, La Rochelle 17000, France; ^3^Esquirol Hospital, Research and Innovation Unit, 15 Rue du Dr Raymond Marcland Limoges, Limoges 87000, France; ^4^Inserm U1094, IRD U270, Limoges University Hospital, EpiMaCT—Epidemiology of Chronic Diseases in Tropical Zones, Institute of Tropical Epidemiology and Neurology, University of Limoges, OmegaHealth, 2 Rue du Dr Raymond Marcland Limoges, Limoges 87000, France; ^5^Chronic Pain Centre, Dupuytren 2 University Hospital, 16 Rue Bernard Descottes, Limoges 87042, France

**Keywords:** agitation, botulinum toxin A, depression, melancholic omega, neuromodulation: amygdala

## Abstract

**Background:** OnabotulinumtoxinA (OnaA) injection in glabella area appears to be a promising treatment for major depression. However, one major concern of placebo-controlled studies on botulinum toxin injections is to ensure adequate blinding.

**Patients and Methods:** In this context, all subjects of this trial received the active product (OnaA). After randomization, 58 patients with resistant major depressive disorder (MDD) received OnaA either in the glabella area (*N* = 29) or in the crow's feet area (*N* = 29). Subjects were blinded to the supposedly effective area against resistant depression and the examiner was not aware of the injected area. The primary outcome measure was the proportion of responders (50% or greater decrease in MADRS [Montgomery and Asberg Depression Rating Scale] score from baseline) in glabella group versus crow's feet group at week 6 after the OnaA injection.

**Results:** The number of responders was significantly higher in the glabella group than in the crow's feet group with 13 responders out of 29 patients (44.8%) in the glabella group and five out of 28 patients (17.9%) in the crow's feet group (*p*=0.029). The rate of psychomotor agitation as measured by item 9 of the Hamilton Depression Rating Scale (HAM-D), associated with a shorter span of psychiatric disorder, was a potent positive predictive factor of positive response to treatment.

**Conclusion:** We conclude that OnaA injected in the glabella muscles is an effective and well-tolerated treatment for MDD. We suggest that patients with a high score at item 9 of the HAM-D might be a subgroup of best responders. We assume that OnaA may act as a modulator of the activity of the primary sensorimotor cortex and then of the amygdala.

**Trial Registration:** ClinicalTrials.gov identifier: NCT03484754

## 1. Introduction

Depression is a common condition worldwide, affecting an estimated 280 million people according to the Institute of Health Metrics and Evaluation (Global Health Data Exchange) [1], source used by the World Health Organization (WHO) [2]. This common illness severely limits psychosocial functioning and diminishes quality of life. It is a major cause of disability worldwide. Major depressive disorder (MDD) is common, with almost one in five people experiencing one episode at some point in their lifetime [3]. An estimated 3.8% of the population is affected, including 5.0% of adults (4% of men and 6% of women) and 5.7% of people over 60 [1, 2]. It affects up to 25% of women and 12% of men during their lifetime and is mostly chronic [4]. Furthermore, the more episodes the patients have, the more likely they are to have additional episodes, subsequently worsening the course of the disorder with every new recurrence. After a first depressive episode, more than 50% of patients have a second episode. The relapse rate increases after each decompensation [5, 6]. Because of the highly prevalent and recurrent nature of depression, its impact is systemic [4].

As many as 30%–40% of patients with MDD are unresponsive to antidepressant medication [7]. Resistance represents a major problem insofar as remission can only be obtained in 35%–40% of cases after a well-conducted antidepressant treatment based on the results of the STAR*⁣*^*∗*^D study, the largest clinical trial conducted in MDD [8]. Resistant depression is a major issue both in terms of public and individual health. It is thought to account for 15%–30% of depressive episodes [9, 10]. Its optimal management is therefore essential. The definition of resistant depression is not always clear in the literature. The current consensus defines it as the failure of two antidepressants of different pharmacological classes, well managed in terms of dosage and duration, while ensuring good compliance (meaning at least 80% of the treatment taken over the period under consideration). Pharmacological treatment can only be considered ineffective after a minimum treatment duration of 4–6 weeks at effective dosage. The optimal minimum duration of antidepressant treatment once the target dose has been reached (consolidation treatment) is 4–6 months for a first episode and can be extended over several years in the case of recurrent depression. Treatment-resistant depressions might play an important role in the medicoeconomic burden of depressive disorders. In 2010, depression ranked second worldwide in terms of disability-adjusted life years [11] due to treatment-resistant depression on the one hand, and to partially responsive depression on the other hand.

Although the number of antidepressants on the market is constantly increasing, as well as the numerous nonmedicinal therapeutic options (psychotherapies, neurostimulation techniques), the development of new treatments for MDD is essential in order to relieve depressive symptoms, particularly in patients with treatment-resistant depression. A new prospect has recently emerged to treat depression. There is a growing body of evidence that modifying the facial expression of sadness and depression with botulinum toxin A might alleviate depressive symptoms [12]. Following the initial description by Finzi and Wasserman [13], seven randomized, placebocontrolled trials have demonstrated the efficacy and safety of the injection of OnaA (Botox, Allergan/AbbVie) into the muscles of the glabellar region (*corrugator* and *procerus*), for treating major depression [14–19]. One major physiological explanation of these results relies on the facial feedback hypothesis (FFH) [12]. According to this hypothesis, frowning is for instance thought to provide sensorimotor feedback to the brain that could contribute to the sensation of a sad emotion. In return, inhibiting frowning in a depressed patient by means of OnaA could interrupt this emotional loop and improve mood.

The most recent meta-analysis assessing the double-blind randomized controlled trials has stressed that the major concern of these studies was the difficulty in ensuring adequate blinding [20], since injecting OnaA into glabella muscles induces visible effects such as impossibility of frowning or reduction of frown lines. Thus, both patient and examiner can guess if the active product has been injected. In an attempt to overcome this concern, we designed our randomized study so that all patients received OnaA, but either in the glabella muscles (*corrugator* and *procerus*) or in the crow's feet muscles (*Orbicularis oculi* [OO]). These two sites of injection were chosen because their injection by OnaA is expected to induce positive genuine effects by diminishing the local facial lines. The control group was the group of patients receiving injections into the OO. The examiner was blind to the site that had been injected and the patients did not know which site was supposed to be effective.

We conjectured that injection of OnaA into the glabella region was more effective than injection into the crow's feet region in reducing depression. The primary objective was to evaluate the efficacy of OnaA injections in the glabella area, in patients with resistant depressive disorder. The control group included patients injected in the OO muscle. We tested the homogeneity between the two groups regarding gender and changes in psychotropic medication between baseline and follow-up visits. The primary outcome measure was the proportion of responders in glabella group versus crow's feet group at 6 weeks after injection. Response was assessed based on changes in the MADRS score between the inclusion visit and the visit 6 weeks after the OnaA injection. For the secondary outcomes, the number of responders and the response based on changes in the MADRS depression score in the glabella group compared with the crow's feet group were examined at 3 weeks and 12 weeks. A difference in effect between the two injection sites on anxiety symptoms, global functioning, and suicidal ideation was observed. Predictive factors were also investigated.

## 2. Materials and Methods

This study was conducted from March 2018 to November 2021 in the regional psychiatric Hospital in Limoges. The duration of follow-up was 3 months per patient. The study was conducted in accordance with the Declaration of Helsinki. All subjects provided written informed consent after a complete description of the study and before their inclusion. We used the CONSORT checklist when writing our report.

### 2.1. Population

Patients were informed of the study during a preinclusion visit, which took the form of an outpatient consultation. All patients signed informed consent before entering the study.

Patients meeting the inclusion criteria were male and female, inpatient or ambulatory patients (18–80 years) with treatment-resistant MDD. MDD was defined according to the criteria of the Diagnostic and Statistical Manual of Mental Disorders Fifth Edition (DSM-V) [21]. Treatment resistance was defined by an absence of symptomatic remission (nonresponse or partial response) in two successive attempts with two antidepressants from different pharmacological classes, well conducted in terms of dosage and duration, while ensuring observance (at least 80% of the treatment taken during the period considered). The attending physician and the psychiatrist responsible for the patient were asked not to change the antidepressant doses from 15 days preceding the injection until 6 weeks after. Women of childbearing age were asked to use an effective method of contraception during the whole study.

Patients were excluded in case of ongoing psychiatric comorbidity (bipolar disorder, psychotic disorder, addictive comorbidity, major neurocognitive disorder), or if they had a contraindication to OnaA treatment (myasthenia gravis, known hypersensibility or infection at the site of injection).

### 2.2. Study Design (Figure 1)

The principle was to inject all patients with the active product, either in the glabella area or in the crow's feet area.

Sociodemographic data (gender, age, family situation, lifestyle, and employment status), medical data (psychiatric diagnosis, personal psychiatric history, family psychiatric history, and presence of somatic comorbidities), patient's treatment at inclusion, and psychometric assessment scales (MADRS, Hamilton Anxiety [HAM-A] scale, item 9 of the HAM-D, Columbia-Suicide Severity Rating Scale [C-SSRS], Global Evaluation of Functioning [GEF] scale, and the Clinical Global Impressions [CGI] scale) were collected at baseline visit.

Eligible patients were randomly assigned to one or other of the groups. Participants were randomized using a randomization list prepared by the Research and Innovation Unit at Esquirol Hospital. OnaA was injected once only at the end of the inclusion visit. The injection of OnaA was carried out by a trained neurologist (DR).

Follow-up visits took place at weeks 3, 6, and 12 after the OnaA injection. At each visit, CCM/the psychiatrist reassessed MADRS, HAM-A scale, C-SSRS, GEF scale, and CGI scale, along with a clinical interview. Muscle relaxation sensation was assessed at week 6.

### 2.3. Study Treatment

In the present study, we sought a procedure that would result in the best control group. The injection of OnaA in the OO muscle is used in aesthetics to reduce crow's feet wrinkles located on the outer part of the eyes. The OO injections seemed to us a satisfying control group. It has, like glabellar injections, a positive cosmetic effect by reducing wrinkles in the corner of the eyes. The lateral part of the OO is not involved in the facial expression of sadness but rather in the facial expression of joy since it contracts during the “true” smile, or Duchenne smile, which involves both the mouth and the eyes [22]. We could therefore expect that by weakening this muscle, there would be no confounding antidepressant effect.

In the present study, all patients received the active product (OnaA), thus the randomization related to the injection site (glabella area versus crow's feet area), the patient being blind to which area was thought to be effective in resistant depression. The examiner (CCM) did not know which area had been injected, the patient was asked not to tell her where he/she had been injected.

A vial of 50 Botox units was diluted in 0.5 ml of 0.9% NaCl solution. This concentration (10 U/0.1 ml) was chosen to avoid as many local side effects, such as ptosis, as possible. Indeed, these side effects are linked to diffusion to adjacent muscles, which is proportional to the employed dilution. Injections were performed using a 30-gauge needle. Depending on the randomization botulinum toxin was injected:

– In the glabella region: 5 units (U) in the *procerus* muscle, 5 units in each *corrugator* muscle.
– In the crow's feet region: 15 units in the OO muscle, in three sites bilaterally.

Safety was assessed through the study.

The primary outcome measure was the proportion of responders in glabella group versus crow's feet group at 6 weeks after OnaA injection. Response was characterized as a 50% or greater decrease in MADRS score from baseline.

### 2.4. Psychometric Evaluations

The following scales were assessed at baseline, weeks 3, 6, and 12:

• The MADRS.
  It is a validated diagnosis questionnaire for hetero-assessment of depressive symptoms in patients suffering from mood disorders. The MADRS includes 10 items (apparent sadness, expressed sadness, inner tension, loss of appetite, concentration difficulties, weariness, loss of feelings, pessimism, and suicidal ideation). Each item has a general definition and six degrees of severity, with degrees 0, 2, 4, and 6 being defined. The higher the score, the more severe the depression. The score can range from 0 to 60. The commonly used subgroups are healthy patients (0–6 points), mild depression (7–19 points), moderate depression (20–34 points), and severe depression (>34 points) [23].
• The HAM-A scale.
  This tool provides a quantitative assessment of anxiety. Its 14 items include all areas of psychological, somatic, muscular and visceral anxiety, cognitive and sleep disorders, and depressed mood. The assessment period goes back to 7 days before the interview. Out of the 14 items, seven measure psychological anxiety (anxious mood, tension, fears, insomnia, cognitive functions, depressive mood, and behavior during the interview) and seven others measure somatic anxiety (general muscular somatic symptoms, general sensory somatic symptoms, cardiovascular, respiratory, gastrointestinal, genitourinary, and autonomic nervous system symptoms). Each of them is assessed according to five degrees of severity, from absence to disabling intensity. The total amount gives an overall score ranging from 0 to 60. The generally accepted threshold for significant anxiety is 20 [23].
• The GEF scale.
  This validated numerical scale (ranging from 0 to 100) is used in psychiatry to assess individual psychological, social, and occupational functioning, on a hypothetical continuum from mental health to illness. It is divided into 10 levels of functioning: scoring the EGF means choosing the level that best reflects the individual's level of functioning. The scale is graduated along a continuum from 1, representing the most ill individual, to 90, representing an individual who is virtually symptom-free (or with very minimal symptoms) and who functions well enough in his or her social or family environment. The scale is divided into nine equal intervals ranging from 1 to 10, 11 to 20, 21 to 30, and so on. Each of the 10 levels of the EGF scale has two components: symptomatic severity and functioning. A score from 0 to 100 is assigned to the patient, taking into account only current psychological, social, and professional functioning [23].
• Item 9 of the HAM-D scale at baseline.
  Item 9 is used to assess agitation. The higher the score, the more intense the agitation [23]. Scoring of agitation: 0: none, 1: twitching, muscle twitches, 2: plays with hands, hair, etc., 3: moves, cannot sit still, and 4: wrings hands, bites nails, pulls hair, and bites lips.
• The C-SSRS.
  This scale is intended to be administered by a trained examiner. The questions in the Columbia Suicide Risk Assessment Scale are suggestions only. In the end, ascertaining the existence of suicidal ideation or behavior results from the judgment of the same examiner [23].
• The CGI scale.
  This scale includes three independent CGI: severity of illness (0–7), global clinical improvement after treatment (0–7), and a composite score on four points that addresses treatment efficiency and adverse effects [23].

### 2.5. Statistical Analysis

*Calculation of the number needed to treat*. Considering a proportion of responders to the treatment of 15% in the group injected in the OO muscles and 50% in the group injected in the glabella area, we needed to include 26 subjects per group (beta power 80%, alpha risk 5%). Taking into account a 10% loss during the follow-up period, it was decided to include 29 subjects per group altogether, that is, a total of 58 subjects.

The variables at baseline and at the different follow-ups were described, for the overall population and for each subgroup. Quantitative variables were described according to mean ± standard deviation, and qualitative variables were described according to numbers or frequencies.

Comparisons of qualitative variables (clinical improvement) between groups at different follow-ups were made using the chi-squared (*χ*^2^) test and the Fisher's exact test when necessary. Scores on psychometric scales between groups at different follow-ups were compared using the nonparametric Mann–Whitney (MW) *U* test. The predictive factors for toxin injection in *corrugator* and *procerus* were searched for with a binomial linear regression test. Modifications of scores at the psychometric scales from one follow-up to another for each of the patients were performed by the Wilcoxon matched pairs tests.

*p*-Values less than 0.05 were considered statistically significant. All analyses were performed using SPSS 27.0.0 for Windows.

## 3. Results

Fifty-eight patients were included, 48 women (82.8%) and 10 men (17.2%), with a mean age of 55.1 ± 11.8 years (min 36–max 78). After randomization, 29 participants received injection in the *corrugator* and *procerus* muscles (glabella group: site thought to show benefit and efficacy after OnaA injection on depressive symptoms) and 29 participants received injection in the OO muscles (crow's feet group: site not thought to show efficacy after OnaA injection on depressive symptoms). Sociodemographic, psychiatric clinical characteristics, psychotropic medication, and psychometric assessments of population are shown in Table 1. They did not differ significantly between the two groups at baseline.

The groups were homogeneous regarding changes in pharmacological treatment during the course of the study, whether between inclusion and week 3 (*p*=1.000), between inclusion and week 6 (*p*=0.352), or between inclusion and week 12 (*p*=0.420). Clinical response was therefore not affected by any change in treatment.

### 3.1. Difference of Effect Between Males and Females

As for the gender, there was no difference (Fisher's test) in the primary outcome between men and women either for the whole group (*p*=1.000) or by injection group (glabella group, *p*=0.573 and crow's feet group, *p*=1.000).

### 3.2. Proportion of Responders at Week 6 (Primary Outcome)

**Figure 2:**
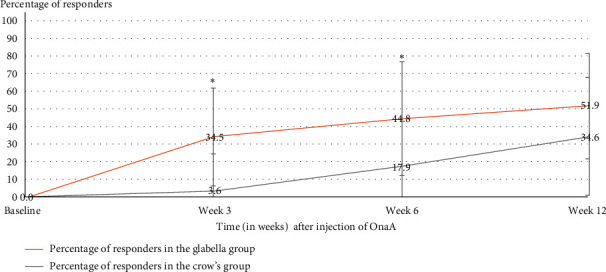
Percentage (%) of responders (according to MADRS score) after injection of OnaA (*t* = 0), in the two groups (*N* = 53) at baseline, 3, 6, and 12 weeks. *⁣*^∗^ Statistically significant (*p*  < 0.05).

The proportion of responders (50% or greater decrease in MADRS score from baseline) at week 6 was significantly higher in the glabella group than in the crow's feet group with 13 responders out of 29 patients (44.8%) at week 6 in the glabella group and five responders out of 28 patients (17.9%) in the crow's feet group (*p*=0.029, *χ*^2^) (Figure 2).

### 3.3. Proportion of Responders in Each Group, 3 and 12 Weeks After the OnaA Injection

At week 3, the difference between the number of responders in the two groups was already statistically significant with 10/29 responders (34.5%) in the glabella group versus 1/28 responders (3.6%) in the crow's feet group (*p*=0.005, *χ*^2^) (Figure 2).

At week 12, there were 14/27 responders (51.9%) in the glabella group and 9/26 (34.6%) in the crow's feet group, which did not reach statistical significance (*p*=0.206, *χ*^2^) (Figure 2).

### 3.4. Evolution of MADRS Score

**Figure 3:**
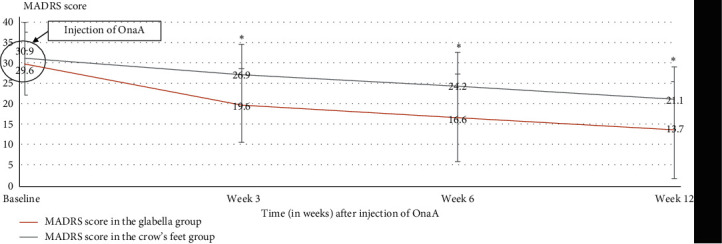
Evolution of MADRS score after injection of OnaA (*t* = 0), in the two groups (*N* = 53) at baseline, 3, 6, and 12 weeks. *⁣*^∗^ Statistically significant (*p*  < 0.05).

From baseline to week 6, the mean MADRS score decreased in both groups, but the glabella group improved more significantly (16.6 ± 8.1) than the crow's feet group (24.2 ± 10.5), and this was statistically significant (*p*=0.006) (Figure 3). Also, there was a significantly greater decrease percentage of the MADRS score in the group receiving the glabella injection (−43.7%) than in the crow's feet group (−26.2%) (*p*=0.025, MW).

At week 3, the MADRS score was significantly lower in the glabella group (19.6 ± 7.4) than in the crow's feet group (26.9 ± 8.9) (*p*=0.001) versus baseline (Figure 3). Also, there was a significantly greater decrease percentage of the MADRS score in the group receiving the glabella injection (−34.5%) than in the crow's feet group (−3.6%) (*p*=0.015, MW).

At week 12, the MADRS score was significantly lower in the glabella group (13.7 ± 7.8) than in the crow's feet group (21.1 ± 11.7) (*p*=0.028) (Figure 3). The percentage reduction in the MADRS score was much greater in the glabella group (−50.9%) than in the crow's feet group (−32.7%); however, this result did not reach statistical significance (*p*=0.051, MW).

### 3.5. Changes in Psychometric Evaluations and Suicidal Ideation 6 Weeks and 12 Weeks After the OnaA Injection in Each Group

At week 6, the GEF scale score significantly improved in the glabella group compared to the crow's feet group (*p*=0.004) (Table 2), as well as at week 12 (*p*=0.006) (Table 3).

The HAM-A scale improved in the glabella group compared to the crow's feet group, but it did not reach statistical significance at week 6 (*p*=0.060) (Table 2), nor at week 12 (*p*=0.059) (Table 3).

At week 6, subjects had no more suicidal ideation in the glabella group than in the crow's feet group (*p*=0.352) (Table 2), neither at week 12 (*p*=0.491) (Table 3).

### 3.6. Predictive Factors of the Efficacy of Botulinum Toxin Injection: Psychomotor Agitation Score on Item 9 of the HAM-D Scale

We explored the variables having a statistically significant discrepancy at 6 weeks in the clinical response criteria in a regression model. Those variables included active smoking (group without improvement: four versus group with improvement: nine, *p*=0.027), span of the psychiatric disorder (group without improvement: 142.00 ± 118.06 months versus group with improvement: 36.62 ± 34.16 months, *p*=0.001) and total score at HAM-D item 9 (group without improvement: 0.13 ± 0.50 versus group with improvement:1.00 ± 0.82, *p*=0.001). After the introduction of these variables into a stepwise binary logistic regression, item 9 score alone from HAM-D alone, or combined with the span of the psychiatric disorder, was predictive of the patient's clinical improvement at week 6.

Interestingly, in the glabella group only, item 9 score alone from HAM-D, or the latter combined with the span of the psychiatric disorder, accounted for 75.8% of the variance of the clinical improvement at week 6. The regression coefficient associated with the span of the psychiatric disorder was significantly negative (*β* = −0.023, *p*=0.019), whereas the regression coefficient associated with item 9 score from HAM-D was significant (*β* = 4.299, *p*=0.024). Thus, a shorter span of psychiatric disorder is associated with a higher psychomotor agitation as shown by a higher HAM-D item 9 score (*p* ≤ 0.001) was the most discriminating combination of predictive factors of clinical improvement at week 6.

### 3.7. Safety

We observed no local side effects such as ptosis, diplopia, or facial asymmetry. The injector (DR) noted that injections into the muscles of the glabella region, but not into the OO muscles, were particularly painful in some patients, with pain evaluated at 10 on a 0–10 numeric rating scale, sometimes requiring the application of ice to alleviate it. The injector (DR) has a 30-year experience in injecting botulinum toxin in glabellar area, either for blepharospasm or for painful conditions such as migraine. She was therefore very surprised by the intensity of that pain. Unfortunately, we did not systematically collect these data.

## 4. Discussion

The present study compared the effects of a single treatment of OnaA injected in the glabella area, considered as a target of choice versus in the crow's feet area, considered as a neutral site.

The primary endpoint was the percentage of responders in glabella group versus crow's feet group at 6 weeks. Response was defined as a reduction of at least 50% in the MADRS score compared with the initial score. We found significant improvement with 44.8% in the glabella group and 17.9% in the crow's feet group. Of note, the results were already significant at week 3 with 34.5% in the glabella group versus 3.6% in the crow's feet group. At week 6 and 12 weeks, we showed a significantly better difference in global functioning as assessed in the glabella group versus the crow's feet group.

Our study confirms previous results regarding the efficacy of botulinum toxin in the treatment of patients with treatment-resistant depression. Injecting botulinum toxin into the glabellar region could therefore be a new, effective, risk-free, and long-lasting treatment option for depression. Up to 30%–40% of patients with MDD do not respond to an attempt at antidepressant medication [7]. According to the results of the STAR*⁣*^*∗*^D study, the largest clinical trial ever conducted for MDD, only 30% of patients with MDD went into remission after 1 year of antidepressant treatment [8]. Even though the number of antidepressant medications and nonpharmaceutical treatment options is increasing, this is still not enough for patients with treatment resistance. As a result, about 70% of patients with MDD require other treatment options to improve the effect of antidepressants. Treatment with botulinum toxin could be one of these options.

These results are in line with those found in six studies conducted by four independent teams using a classical placebo-controlled design [14–19]. In the study by Wollmer et al. [14], the response rate was significantly higher in the group receiving active treatment. Finzi and Rosenthal [16] found a response rate at 6 weeks after injection of 52% in the OnaA group versus 15% in the placebo group. The cross-over study by Magid et al. [15] also showed that patients who received OnaA first and those who received it at 12 weeks had a significantly higher response rate.

In addition, in our study, a significant reduction of the MADRS score at week 6 (*p*=0.006) was shown. Here again, these data are consistent with the results of previous randomized studies. As a reminder, the reduction of depression score was 47.1% in the OnaA group versus 9.2% in the placebo group in Wollmer et al.'s [14] study, 47.3% versus 20.6% in Finzi and Rosenthal's [16] study, and 46% versus 2% in Magid et al.'s [15] study.

So far, the GEF scale has not been used in previous studies. The GEF is a complementary scale to the MADRS as it makes it possible to assess the effect of botulinum toxin on the patient's function, which was not considered in previous studies. This significant difference between the two groups is an innovative and useful aspect of this scale.

Our study design aimed at overcoming the issue of inadequate blinding when injecting OnaA in muscles of the face. Indeed, in a placebo-controlled design, patients may be aware that the active product has been injected, as they cannot frown anymore. The previous studies faced this difficulty of ensuring double blindness as the control group was injected with a saline placebo [14–19]. That is why, we chose to use the active product, that is, OnaA, in both groups but to mask the site of injection to the examiner. In this perspective, glabella was considered as the active site and crow's feet site as the neutral/control site. Patients did not know which site was supposed to be active, and the examiner did not know where the patient had been injected. The validity of our paradigm relies on the fact that the crow's feet site is indeed a neutral site. The lateral part of the OO is involved in the facial expression of joy since it contracts during the “true” smile or Duchenne smile, which involves both mouth and eyes [22]. We could therefore expect that, by weakening OO by means of OnaA injection, there would not be any antidepressant effect. Conversely, Lewis [22] suggested in that OnaA injections into OO could induce a depressed mood. The author compared the score on the Hospital Anxiety and Depression Scale (HADS) between subjects injected in the glabella only and those injected in both glabella and crow's feet. The HAD scale score was significantly reduced in the first group, but not in the second group. The author concluded that the botulinum toxin injection in OO may be responsible for this discrepancy. However, this study has some important biases. Few subjects were included, and they were healthy subjects and not depressed patients. Moreover, this study was not designed to investigate this issue and such an indirect comparison cannot prove that injection of botulinum toxin into the OO may have deleterious effects on mood. Our results rule out this hypothesis, since the patients in the crow's feet group did not have an aggravation of their depressive symptoms during the study. We thus think that the crow's feet area constitutes a suitable control group for applying OnaA in depressed patients.

It was possible to characterize the predictive factors of this clinical response using a regression model. We have prospectively assessed the predictive value of high psychomotor agitation at initial evaluation and after OnaA. We found that a higher psychomotor agitation at baseline (as measured by item 9 of the HAM-D) was a predictor for better response to OnaA. A shorter span of depressive disorder associated with a higher psychomotor agitation was the most discriminating combination of predictive factors for clinical improvement at week 6. Wollmer et al. [14], in a post hoc analysis of their initial study, also found that a higher baseline level of psychomotor agitation had a good positive predictive value [24]. Thus, we could consider that a high psychomotor agitation score at inclusion could lead practitioners to choose botulinum toxin in the case of resistant depression rather than a change in antidepressant drug or treatment with neuromodulation (rTMS [repetitive transcranial magnetic stimulation] and ECT [electroconvulsive therapy]). A 2021 study on factors predicting clinical response after rTMS for patients suffering from resistant depression showed that the combination of a more recent depressive disorder and a higher psychomotor agitation score (assessed with item 9 of the HAM-D) at inclusion was also a factor in a good response to rTMS treatment, explaining 69.2% of the variance in clinical improvement [25]. In our study, OnaA as a nondrug therapy showed even better results. Interestingly, Greden, Genero, and Price [26] showed that psychomotor agitation in MDD patients was correlated with increased electromyogram activity in the *corrugator* muscle region. When both *corrugator* and *procerus* contract, they form vertical lines between the eyebrows, which, conjugated with contraction of frontalis muscle, form a wrinkle (between the eyebrows) in the shape of the last letter of the Greek alphabet, the “omega.” It may be suggested that the melancholic omega melancholium could be a clinical marker of psychomotor agitation in depressed patients. It should be interesting to investigate whether the presence of this clinical sign might also be a predictive factor of clinical response to OnaA.

The concentration and total amount of injected botulinum toxin is an important issue to consider. We used 15 U in the glabella, whether the patients were male or female. This dosage is derived from the one generally used for treatment of blepharospasm. In previous studies on depression, women generally received 29 U and men 39 U. In the Allergan study, it is striking to note that the group receiving 50 U globally underwent less improvement than the 30 U group. Such observations were reported in the first steps of development of the use of OnaA in migraine, where 25 U of OnaA injected pericranially were more effective than 75 U [27]. It illustrates that we do not know dose–response relationship for OnaA for most of its applications [28], *a fortiori* for emerging indications such as depression. As a matter of fact, using lower doses of OnaA has the benefit of making its motor effects less visible. Consequently, we can assume that this also reduces the placebo effect, as predicted by Arnone et al. [20].

The biochemistry of OnaA is to inhibit acetylcholine exocytosis at the neuromuscular junction [29]. The exact mechanism by which OnaA may act on depression remains unknown. Preclinical studies have shown that peripherally injected OnaA may induce biochemical modifications in cerebral structures involved in depression [30]. In a rat model of orofacial pain, the facial application of Botulinum Toxin A caused a significant increase of noradrenalin in the striatum and of serotonin in the hypothalamus [30]. In a mice model of depressive-like behavior, Botulinum Toxin A injection significantly increased serotonin (5-HT) levels in several brain regions, including the hippocampus and hypothalamus, and significantly increased the expression of brain-derived neurotrophic factor (BDNF) in the hippocampus [31]. Moreover, recent in vivo data suggest that there is a functional connection between the *corrugator* muscles and the amygdala [32], a cerebral structure which is known to be central to the pathogenesis of MDD [33]. This relationship is reciprocal. Intracerebral stimulation of the amygdala in human increases *corrugator* muscle activity [32]. In return, denervation of frown muscles by means of Botulinum Toxin modulates amygdala activity, as demonstrated by functional magnetic resonance imaging (fMRI) [34, 35]. It can be hypothesized that in depressed patients, OnaA decreases the abnormal, excessive motor/proprioceptive input arising from the glabellar muscles onto the brain and regulates the functioning of the affective brain. However, this hypothesis requires further neurobiological evidence. Brain neuromodulation by Botulinum Toxin A has yet to be demonstrated in classical indications such as cervical dystonia [36] and is in line with the FFH.

We are aware of the limitations of our study.

This trial is monocentric. The sample size was calculated based on a hypothesis of 50% responders in the treatment group and 15% responders in the placebo group at week 6, which was almost met (44.8% and 17.9%, respectively). Therefore, the statistical power of this study is limited but significant and needs to be confirmed by other multicentric studies on a larger scale. It is one of the largest studies to date on this topic. If the number of participants had been higher, we could have expected the variable duration of disease to be more homogeneous and to maybe be an isolated predictive factor of the improvement at week 6.

We did not investigate pain at injection, which seemed severe for certain patients. If confirmed, it could indicate an abnormal sensory processing in certain depressed patients.

Although our study included a small number of men, the groups were homogeneous in terms of gender. The generalization of our results to the male population should be treated with caution. However, the greater inclusion of women, as in all previous trials, can certainly be explained by the epidemiological fact that depression affects women more than men. Women are more vulnerable to depression and resistance to treatment. However, this alone cannot explain the significant disproportion in the sex ratio.

Furthermore, even if the randomized baseline population injected at two different sites with botulinum toxin shows no significant difference regarding the duration of the disorder, the univariate analysis preceding regression showed that the group with improvement had a less duration of illness than the group without improvement. Although the short duration of psychiatric illness appears to be the most influential factor in participant improvement based on statistics, these data do not stand alone during regression, unlike psychomotor agitation, likely due to the associated large standard deviation. Conversely, as discussed, the combination of both elements, namely the duration of the disorder and the psychomotor agitation, seems to be of significant interest as they account for improvement together in the majority of cases. Several clinical trials have already shown that the duration of the depressive disorder is inversely correlated to the efficiency of the treatment on trial [37–40]. This combination aligns with what has been demonstrated with nonpharmacological rTMS treatment as predictive factors for participant improvement in cases of treatment-resistant depression [25]. Therefore, psychomotor agitation appears to be more important and innovative in this equation of participants' improvement but it must be considered in light of the duration of participants' psychiatric illness for further studies.

Further research should consider focusing on the predictive factors by creating homogeneous subgroups for the depressive illness (length of time and duration of the disorder, severity). This could be introduced into the study's inclusion and exclusion criteria.

Further studies are also warranted to determine how OnaA could be incorporated into treatment algorithms for depression, at least as an additional adjuvant treatment, to prevent relapse, in treatment resistance, or in cases of intolerance to antidepressants. If future larger randomized controlled trials were to confirm its efficiency and its better results when prescribed earlier in the duration of the depressive disorder, OnaA could even be considered as a single treatment trial in monotherapy, considering its minor side effects.

Treatment by injection of botulinum toxin into the glabellar region could become a new therapeutic option and be a part of the strategies for potentiating antidepressant treatment in the management of resistant depression, especially if future studies confirm the safety and efficacy data from studies conducted to date.

## 5. Conclusions

We conclude that injection of OnaA into the glabella muscles is a new, effective, risk-free, and long-lasting treatment option for resistant depression. Our study confirms previous results regarding the efficacy of botulinum toxin in the treatment of patients with treatment-resistant depression. Further studies are needed with larger scale population. Future research could also benefit from longer follow-ups, more gender-balanced samples, and larger scale population to confirm these preliminary results. Another issue is to find a proper place for this treatment in the pharmacopea of depression. It seems necessary to target endophenotypes of depression susceptible to respond better to OnaA. Patients with a high score at item 9 of HAM-D might be a suitable subpopulation. We also suggest that OnaA may act as a modulator of the activity of the primary motor cortex and then of the amygdala.

## Figures and Tables

**Figure 1:**
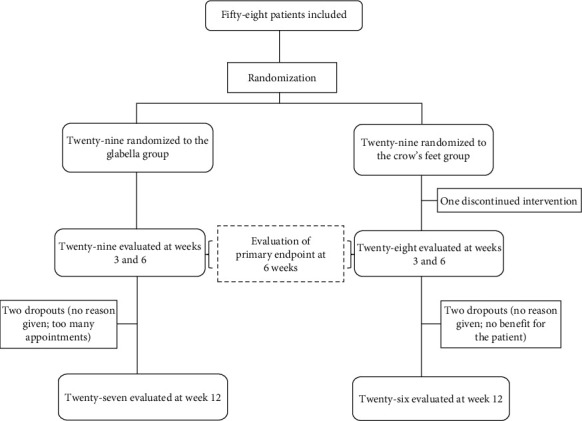
Flowchart.

**Table 1:** Comparative analysis of sociodemographic, psychiatric clinical characteristics, psychotropic medication of population, and psychometric assessments at baseline (*N* = 58).

Variables	Total (*N* = 58)	Glabella group (*N* = 29)	Crow's feet group (*N* = 29)	Significance (between groups)
Demographic data				
Gender (*N* [%])				
Women	48 (82.8%)	26 (89.7%)	22 (75.9%)	*p*=0.297
Men	10 (17.2%)	3 (10.3%)	7 (24.1%)	
Age (mean ± SD)	55.1 ± 11,8	55.0 ± 12.0	55.3 ± 11.8	*p*=0.919
Family situation (*N* [%])				
Single	12 (20.7%)	8 (27.6%)	4 (13.8%)	*p*=0.194
Married/civil union	34 (58.6%)	13 (44.8%)	21 (72.4%)	
Widower	4 (6.9%)	3 (10.3%)	1 (3.4%)	
Divorced	8 (13.8%)	5 (17.2%)	3 (10.3%)	
Lifestyle (*N* [%])				
Living with parents	1 (1.7%)	0 (0%)	1 (3.4%)	*p*=0.027
Single	16 (9.3%)	11 (37.9%)	5 (17.2%)	
Alone with child(ren)	6 (10.3%)	5 (17.2%)	1 (3.4%)	
Couple without children	20 (34.5%)	5 (17.2%)	15 (51.7%)	
Couple with child(ren)	15 (25.9%)	8 (27.6%)	7 (24.1%)	
Professional status (*N* [%])				
Active	11 (19.0%)	6 (20.7%)	5 (17.2%)	*p* =0.931
Inactive	28 (48.3%)	14 (48.3%)	14 (48.3%)	
Retired	19 (32.8%)	9 (31.0%)	10 (34.5%)	
Psychiatric clinical characteristics at inclusion				
ICD-10 diagnosis (codage)				
F32.0	1	1	0	*p* =0.464
F32.1	16	7	9	
F32.2	18	11	7	
F32.9	2	0	2	
F33.0	5	2	3	
F33.1	4	3	1	
F33.2	9	3	6	
F33.9	3	2	1	
Personal psychiatric history (*N* [%])	46 (79.3%)	24 (82.8%)	22 (75.9%)	*p*=0.517
History of suicide attempt (*N* [%])	20 (34.5%)	13 (44.8%)	7 (24.1%)	*p*=0.097
Psychiatric family history (*N* [%])	39 (67.2%)	19 (65.5%)	20 (69.0%)	*p*=0.780
Duration of current depressive episode (mean ± SD, months)	12.5 ± 12.6	11.6 ± 10.2	13.5 ± 14.7	*p*=0.827
Duration of the depressive disorder (mean ± SD, months)	110.8 ± 109.2	94.8 ± 104.0	126.9 ± 113.8	*p*=0.101
Presence of suicidal ideation (*N* [%])	14 (24.1%)	7 (24.1%)	7 (24.1%)	*p*=1.000
Psychotropic medication at inclusion (*N* [%] of patients)				
Antidepressant(s)				
One antidepressant	56 (96.6%)	29 (100%)	27 (93.1%)	*p*=0.491
Two antidepressants	27 (46.6%)	16 (55.2%)	11 (37.9%)	*p*=0.188
SSRI(s)	23 (39.7%)	15 (51.7%)	8 (27.6%)	*p*=0.060
SNRI(s)	20 (34.5%)	10 (34.5%)	10 (34.5%)	*p*=1.000
Other	25 (43.1%)	11 (37.9%)	14 (48.3%)	*p*=0.426
Tricyclic	2 (3.4%)	1 (3.4%)	1 (3.4%)	*p*=1.000
MAOIs	6 (10.3%)	3 (10.3%)	3 (10.3%)	*p*=1.000
Anxiolytic(s)	44 (75.9%)	23 (79.3%)	21 (72.4%)	*p*=0.539
Hypnotic(s)	24 (41.4%)	13 (44.8%)	11 (37.9%)	*p*=0.594
Antiepileptic(s)	17 (29.3%)	11 (37.9%)	6 (20.7%)	*p*=0.149
Antipsychotic medication(s)				
One antipsychotic	26 (44.8%)	14 (48.3%)	12 (41.4%)	*p*=0.597
Two antipsychotics	9 (15.5%)	5 (17.2%)	4 (13.8%)	*p*=1.000
Psychometric assessments (mean ± SD)				
MADRS	30.3 ± 8.1	29.6 ± 8.7	30.9 ± 7.5	*p*=0.293
HAM-A scale	25.7 ± 7.6	24.4 ± 7.0	27.1 ± 8.0	*p*=0.224
GEF scale	48.0 ± 11.2	48.2 ± 10.9	47.8 ± 11.7	*p*=0.851
GCI scale	4.8 ± 0.9	4.8 ± 0.9	4.9 ± 0.9	*p*=0.863

Abbreviations: F32.0, mild depressive episode; F32.1, moderate depressive episode; F32.2, severe depressive episode without psychotic symptoms; F32.9, depressive episode, unspecified; F33.0, recurrent depressive disorder, current episode mild; F33.1, recurrent depressive disorder, current episode moderate; F33.2, recurrent depressive disorder, current episode severe without psychotic symptoms; F33.9, recurrent depressive disorder, unspecified; MAOI(s), monoamine oxidase inhibitor drugs; *N*, number; SD, standard deviation; SNRI(s), selective serotonin and noradrenaline reuptake inhibitor(s); SSRI(s), selective serotonin reuptake inhibitor(s).

**Table 2:** Changes in psychometric characteristics and suicidal ideation between baseline and week 6, in the glabella group, and in the crow's feet group.

Variables	Glabella group (*N* = 29)	Crow's feet group (*N* = 28)	Significance *p* (between the two groups)
HAM-A scale score (mean ± SD)			
Baseline	24.4 ± 7.0	24.4 ± 7.0	*p*=0.224
Week 6	17.5 ± 7.3	22.2 ± 9.0	*p*=0.060
GEF scale score (mean ± SD)			
Baseline	48.2 ± 10.9	47.5 ± 11.8	*p*=0.851
Week 6	62.5 ± 11.7	53.0 ± 11.9	*p*=0.004*⁣*^*∗*^
Number of subjects with suicidal ideation			
Baseline	7	7	*p*=1.000
Week 6	1	3	*p*=0.352

*⁣* ^∗^ Statistically significant (*p*  < 0.05).

**Table 3:** Changes in psychometric characteristics between baseline and week 12, in the glabella group, and in the crow's feet group.

Variables	Glabella group (*N* = 27)	Crow's feet group (*N* = 26)	Significance *p* (between the two groups)
HAM-A scale score (mean ± SD)			
Baseline	25.0 ± 6.9	27.2 ± 8.3	*p*=0.224
Week 12	14.9 ± 7.6	20.3 ± 10.3	*p*=0.059
GEF scale score (mean ± SD)			
Baseline	48.0 ± 11.3	47.5 ± 12.2	*p*=0.851
Week 12	65.4 ± 12.1	56.1 ± 12.8	*p*=0.006*⁣*^*∗*^
Number of subjects with suicidal ideation			
Baseline	7	7	*p*=1.000
Week 12	0	1	*p*=0.491

*⁣* ^*∗*^ Statistically significant (*p*  < 0.05).

## Data Availability

The data presented in this study are available from the corresponding author upon request.
